# Challenges of the Pandemic Response in Primary Care during Pre-Vaccination Period: A Qualitative Study

**DOI:** 10.1186/s13584-015-0028-5

**Published:** 2015-10-15

**Authors:** Marina Kunin, Dan Engelhard, Shane Thomas, Mark Ashworth, Leon Piterman

**Affiliations:** Melbourne School of Population and Global Health, the University of Melbourne, Melbourne, Australia; Paediatric Department, Hadassah-Hebrew University Hospital, Jerusalem, Israel; Office of Pro Vice Chancellor International, the University of Adelaide, Adelaide, Australia; Department of Primary Care and Public Health Sciences, King’s College London, London, UK; Office of the Deputy Vice-Chancellor (Education), Berwick & Peninsula, Monash University, Melbourne, Australia

**Keywords:** Primary Health Care, Pandemics, Disease outbreaks, Public Health, Qualitative research, Health politics, General practice, Influenza, H1N1, Preparedness

## Abstract

**Background:**

During the 2009/A/H1N1 pandemic, the main burden of the patient management fell on primary care physicians (PCPs), and they were the principal implementers of pandemic policies. Broad involvement of PCPs in the pandemic response offered an excellent opportunity to investigate the challenges that they encountered.

**Objective:**

To examine challenges faced by PCPs as they implemented pandemic policies in Australia, Israel and England before the 2009/A/H1N1 pandemic vaccine became available.

**Methods:**

This is a qualitative descriptive study that employed in-depth semi-structured interviews with 65 PCPs from Australia, Israel and England. The data were analysed thematically to provide a detailed account of the themes.

**Results:**

Challenges in three fields of the pandemic response were identified. (i) Consultation of patients was challenged by the high flow of patients, sick and worried-well, the necessity to provide personalised information about the disease during consultations, and unfamiliar antiviral treatment. (ii) Performance of public health responsibilities was complicated in regards to patient segregation and introduction of personal protection measures. (iii) Communication with the health authorities was inefficient, with no established route to provide feedback about the pandemic policies.

**Conclusions:**

The experience of the 2009/A/H1N1 pandemic highlighted the centrality of primary care in the pandemic response. Despite intensive pre-pandemic planning, numerous barriers for implementation of the pandemic policies in primary care were identified. Investigation of three different approaches for involvement of PCPs in the pandemic management showed that none of these approaches worked smoothly.

## Background

During the 2009/A/H1N1 pandemic, the main burden of managing the patients fell on primary care physicians (PCPs) [1, 2], and they were the principal implementers of pandemic policies. Broad involvement of PCPs in the 2009/A/H1N1 pandemic offered an excellent opportunity to investigate the challenges that PCPs encountered and to improve preparedness plans.

This study aimed to examine challenges faced by PCPs as they implemented pandemic policies in three countries, Australia, Israel and England. It investigated the views of PCPs (General Practitioners (GPs) in Australia and England), who consulted patients before the 2009/A/H1N1 pandemic vaccine became available. The rationale for selection of these countries was based on facts that all three countries have a high standard of public health, universal coverage for health services, and high health care accessibility [3–5]. All three countries were in a state of high pandemic preparedness, had developed pandemic response plans, stockpiled personal protective equipment (PPE) and antiviral drugs [6–8]. The existing research links in these countries were also an important factor needed to facilitate the logistics of data collection.

Despite stated similarities, linkages between PCPs, other ambulatory health services, hospitals and Health Departments in these three countries vary depending on decentralisation of the health system, its financing, and relationship with regulatory and legal system [3–5]. This difference in the level of decentralization of the primary care services is expected to ensure greater generalizability of the findings.

### Three approaches to involvement of PCPs in the pandemic response

When the World Health Organization declared a “significant increase in risk of a pandemic”[9], each of the three countries employed strategies to delay the appearance of the disease and to contain its spread. During this containment phase, a range of policy measures, such as active epidemiological investigation, isolation of cases and school closure, were used. This period, from the first confirmed case of the pandemic flu until the official announcement about the change in the response strategy, lasted 4 weeks in Victoria, 8 weeks in Israel and 9.5 weeks in England. Each of the three selected countries employed different strategies for the involvement of PCPs during this period [10].

The Israeli approach during the containment phase was to protect PCPs and the public directing all suspected cases to the hospitals where they were tested and treated [10]. PCPs started to be fully involved in the response once the disease became spread in the community. At that time, it was obvious that the disease was not as severe as previously thought.

In both Australia and England, PCPs were at the front line from the outset of the pandemic, expected to both test and treat all suspected patients [10].

In Australia, PCPs were in charge of preparing a triage plan for suspected cases and introducing infection control measures in their clinics, testing, prescribing antiviral treatment and reporting the suspected cases to the health authorities [10]. Their role was intensive and constant throughout the pandemic period.

In England, the approach was to prevent primary care clinic attendance by the suspected patients [10]. Throughout the containment phase, most suspected patients were assessed by PCPs during home visits. Then, advice for self-treatment and prescription of antivirals through National Pandemic Flu Service (NPFS) was provided. People, who fulfilled the criteria according to the symptom-based clinical algorithm, were given an authorisation code that a ‘flu friend’ – someone who did not have H1N1 – could use to collect antivirals. Only at-risk and patients with severe symptoms were advised to consult PCPs so that the majority were kept out of primary care clinics [10].

## Method

This is a qualitative descriptive study [11] that employed in-depth semi-structured interviews with PCPs to understand their experience from their own perspective [12].

### Sampling

The sampling strategy was directed towards recruitment of “information-rich” cases [13]:

- PCPs who practiced in areas with substantial 2009/A/H1N1 activity and/or started to consult the 2009/A/H1N1 patients early in the pandemic outbreak.
- PCPs who were more involved in implementing practice policy (for example, PCPs who directed the response of their practice to the pandemic).

#### Participant recruitment in Australia

The first group of doctors was selected from PCPs in Melbourne. PCPs were recruited using the School of Primary Health Care at Monash University research links with the Divisions of General Practice in Melbourne. An invitation to participate in the research was published in the newsletters of the Divisions of General Practice in Melbourne. In addition, a number of PCPs were recruited using the “snowball” sampling method [14] when the participants indicated PCPs who might be interested to participate in the research.

#### Participant recruitment in Israel

The second group was selected from the primary care physicians in Israel using the research links of Hadassah Hospital with the Organization of Family Practitioners and the Organization of Child Practitioners in Israel. One of the authors (DE), advised on potential participants based on the familiarity with PCPs who directed the pandemic response of their practice and information about areas with substantial 2009/A/H1N1 activity. PCPs were contacted over the phone by one of the authors (MK) and, after an explanation about the research, were invited to participate.

#### Participant recruitment in England

The third group was selected from the PCPs in London using Kings College research links with PCPs of Lambeth and Southwark Primary Care Trusts (PCTs) in London. The research was advertised in the surgeries of these two PCTs with the support of one of the authors (MA). PCPs, who expressed interest in the research, were contacted over the phone or in person by one of the authors (MK) and, after an additional explanation about the research, invited to participate.

### Interviewing

All interviews were conducted in a timeframe as close as possible to the 2009/A/H1N1 pandemic to improve data quality and to avoid recall bias (Table 1).

**Table 1:** Time and place of the qualitative data collection

Place of data collection	Australia (Melbourne)	Israel (Central, Tel-Aviv and Jerusalem districts)	England (London)
Ethics Approval	Monash University Ethics Committee	Hadassah Hospital Ethics Committee	South West London Research Ethics Service and NHS Lambeth and Southwark.
Abbreviation for representative quotations	M, M(p) for pilots	I	L
Time of data collection	June, August and September 2010	July 2010	July 2010
Number of interviewees	20 - main study 5 - pilots	20	20

All interviews lasted 30–45 min and the schedule included a standard set of topics presented by the interviewer (MK), which included:

- Clinic responses in order to manage the A/H1N1 2009 pandemic
- Expectations from the health authorities
- PCPs’ view on the role of the primary care during a pandemic outbreak
- Successes and challenges in managing the 2009/A/H1N1 pandemic.

These general topics were developed into specific questions in the process of the discussion. The current report includes only data that were prompt by questions about the pre-vaccination period.

### Data analysis

The data were transcribed verbatim and analysed thematically to provide a detailed account of the themes. The analysis was informed by the six-phase approach of Braun and Clarke [15]. Organization of the data was assisted through the computer program NVivo9.

After the initial familiarization with the data through reading of the complete interview transcripts, initial codes were generated by the first author (MK). This analytical approach was inductive, which allowed the codes to be developed “on the go”. In this way, a new code was created every time a feature of the data appeared which was relevant to the research question as the researcher systematically worked through the data giving equal attention to each data item [16]. The initial coding was accompanied by the “memoing” process of writing remarks about the data, questions for further clarification and theorising. The memos were used for later reflection about the data.

When the coding was completed, the categories were reviewed by three of the authors (MK, LP and DE), refined and three broader categories that describe PCPs activities were agreed upon.

## Results

Sixty five PCPs were interviewed for the study. Characteristics of the study sample are presented in Table 2.

**Table 2:** Sample characteristics

Place of data collection	Australia (Melbourne)	Israel (Central, Tel-Aviv and Jerusalem districts)	England (London)
Gender:			
Male	16	12	8
Female	9	8	12
Age:			
Less than 30	0	0	2
30–39	1	5	12
40–49	5	5	3
50–59	15	7	3
60–69	4	3	0
Year of practice:			
Less than 5	1	2	12
5–10	1	5	2
11–20	3	4	1
21–30	15	9	5
More than 30	5	0	0
Total N	25	20	20

In organizing data thematically, 7 themes emerged as the main concerns of PCPs with the burden on primary care during the pre-vaccination period of the pandemic. These themes were further grouped under three broad categories (Table 3).

**Table 3:** Themes emerged from the data

1. Patient consultations	1.1 high flow of flu patients and worried-well
	• surges of people who were concerned they may get sick
	1.2 provision of information
	• patients seeking personal reassurance
	1.3 antiviral treatment
	• limited experience in prescribing antiviral drugs (Oseltamivir (Tamiflu) and Zanamivir (Relenza))
	• prescribing according to strict guidelines
2. Public health responsibilities	2.1 patient segregation
	• difficulty of isolating flu patients in primary care clinics
	2.2 personal protection
	• convenience of using PPE in primary care
	• supply of PPE
3. Communication with the health authorities	3.1 communication of policies and guidelines to PCPs
	• redundant and conflicting communication from multiple sources
	• guidelines - frequently updated, lengthy, not oriented to primary care, conflicting with on-the ground experience
	3.2 bottom-up communication from PCPs
	• time consuming reporting to the authorities
	• no route to provide feedback about the on-the-ground experience to the authorities

### Patient consultations

PCPs reported that patient consultations included an assessment, provision of antiviral or supportive treatment, reassurance of worried-well and provision of information in the form of personalised advice.

#### High flow of influenza patients and worried-well

PCPs were unanimous that management of worried-well patients was one of their key roles during the 2009/A/H1N1 pandemic. They described media induced anxiety which imposed resource constraints on primary care. The level and type of difficulties PCPs encountered were different in the three countries.

In Australia, where the spread of the pandemic appeared early, PCPs struggled to manage the patients concurrently performing public health responsibilities of reporting suspected cases to the health authorities, making special arrangements for swabbing and antiviral treatment:

*“…we were juggling all these delivery issues, approval process issues, lab results…we tried as best we could…”(M2)*

In England, PCPs described how assessing suspected cases during home visits put a strain on their work during the early stage of the pandemic:

*“…we were expected to visit everybody at home and take swabs with masks and gowns which was hideously impractical.” (L12)*

English PCPs noted a decrease in the flow of patients when NPFS was launched but reported assessing numerous patients who sought reassurance after having consulted the NPFS:

*“…it (NPFS) was so algorithmic that a lot of our patients came back and… we’d have to have another conversation.” (L11)*

English PCPs expressed their support for the NPFS organization; however, concerns with the safety of the NPFS phone consultations were raised. In particular, the fact that the NPFS was staffed with “*non-clinically trained people making decisions to treat people with drugs”(L17)*. PCPs pointed to the fact that “*telephone consultations… are difficult entities for GP’s themselves”(L11)*, which require experience in making a correct diagnosis without seeing a patient.

In Israel, the same level of anxiety did not concur with the spread of the disease in the community. The flow of worried-well ended before the 2009/A/H1N1 patients started to appear in primary care in increased numbers:

*“At the beginning patients were very anxious because of the reports about deaths… but at the peak of the second wave… it was clear that here it is not so bad… so it was less anxiety..” (I17)*

#### Provision of information

PCPs were the main channel for delivering the pandemic policies to patients, usually in a form of specific clinical advice and treatment. Although the health authorities in the three countries made a considerable effort to provide information about the disease, infection control and treatment to the public, PCPs saw themselves as *a “natural source of information for the patients” *(I14).

In Australia, many PCPs reported that the provision of information took a great amount of time, taking into consideration the anxiety of the patients:

*“The vast majority just needed checking out and reassurance… I went over how flu is very different clinically from a bad cold, and symptoms for both, millions of times.” (M7)*

In England, where the NPFS was providing advice over the phone, PCPs still reported surges of patients who were seeking PCP advice. Many PCPs reported that the main concern of the patients in this respect was the applicability of the general information, available through the NPFS and mass media, to their specific situation:

*“… I think most of our patients did request advice even if there was kind of general published advice around. I think they valued speaking to someone, so we did have a large influx at that time, and that continued even after the telephone line (NPFS) came on line.” (L11)*

In Israel, many PCPs also reported the quest for personalized advice from their patients:

*“People came in and asked a lot of practical questions: … what about my mum, what about my dad, my neighbour got it what if the kids will be exposed?” (I14)*

#### Antiviral treatment

In Australia and England, where the first cases were treated in primary care during the initial period of the pandemic, strict guidelines provided for prescription of the antivirals created clinical practice difficulties. PCPs in Australia and England were required to seek permission from the health authorities to prescribe the drug and in this respect they felt that the health authorities did not rely on their clinical judgment:

*“There were rules (to prescribe the drug), but I think this is where the government’s got to realise that GPs (PCPs) are not stupid. I am sure they (PCPs) are going to usually make decisions in the best interest of the population… They (government) don’t have any confidence in the people that treat people all the time.” (M3)*

Many GPs from each of the three countries reported that they did not use antiviral drugs to treat flu patients prior to the 2009/A/H1N1 pandemic and were generally unfamiliar with these drugs (Tamiflu and Relenza). This contributed to their confusion regarding the treatment and concerns with its safety and necessity:

*“I don’t believe in it… I’ve got an impression that it (Tamiflu) was advised to pacify the public: get Tamiflu and everything will be all right. Because it is hard to say that there is a disease that has no treatment…” (I8)*

*I’ve never prescribed Tamiflu until the swine flu season … it was a bit nerve wracking, because you’re prescribing a drug you don’t really know much about, new territory, you don’t know the risks, you don’t know the pros, and it was a bit unsettling.” (L18)*

### Public health responsibilities

In addition to consulting patients in increased numbers, PCPs had to perform public health responsibilities that were out of scope in their usual clinical routine. These responsibilities included segregation of patients and application of personal protection measures in order to reduce the disease transmission. Many PCPs felt that these public health responsibilities were imposed on them without an appropriate consultation process and that the health authorities did not acknowledge that their role as PCPs *“is different to the role of the public health officer” (M17).* While PCPs are a *“person’s advocates” (L4), “public health policy is specifically geared not to consider individual patient needs”(M17).*

#### Patient segregation

Many PCPs reported that it was impossible to separate patients in a busy clinic. Even in a situation where a spare room for isolation was available, PCPs felt that it was not good practice to have several patients simultaneously in an isolation room, before assessing them for the 2009/A/H1N1.

In Australia, a common practice to separate the suspected flu patients was to assess them in their car or to ask them to wait in a car instead of in a waiting room until a PCP was ready to consult them.

*“..we asked people to ring ahead, we asked if they had a fever or any symptoms of flu, … to stay in their cars… And we would usually give them a mask to walk them in, and walk them straight into the consulting room…” (M7)*

In England, the approach was to keep the suspected patients out of practices and to assess them during home visits or phone consultations. It seems that many patients with the flu symptoms were turned away from the primary clinics, but the practice of putting them into an isolation room was not uncommon:

*“…we tried to encourage people with flu to stay at home as per the guidelines, but when people were coming, to try and have separate rooms that we could …put them in and segregate them.” (L14)*

In Israel, in some Health Maintenance Organization (HMO) clinics that assessed flu patients for a limited period at the end of the containment phase, an effort was made to separate flu patients. Regular primary clinics in Israel started to consult pandemic flu patients when the disease was widely spread in the community, and there was overall acceptance of the fact that the separation was not feasible:

*“When the disease had turned into a pandemic there was no sense in separation, it was impossible.” (I15)*

GPs from the three countries reported that policies introduced to low disease transmission in a clinic (separation of patients in clinics in Australia, assessment in the hospital during the containment phase in Israel, and home visits or phone consultations in England), were not always successful because of the lack of cooperation from patients. A considerable number of patients did not report flu symptoms till they got to see the doctor, thus mingling with other patients in the waiting room:

*“Well naturally we had people that came in … despite all the publicity or despite people telling them or pretending they didn’t know they had the flu, so we did have people that did come in… The problem is they’d probably been sitting in the waiting room for half an hour before they got to your room and before you realised.” (L14)*

#### Personal protection

PCPs from all three countries described that PPE, such as masks, gloves and gowns, did not *“make for good patient-doctor communication or rapport”*(L18), and their use was time-consuming. The main difficulty was the necessity to change PPE between consultations. Many PCPs reported that they ceased using PPE after a number of attempts due to the inconvenience of use:

*“… I can’t change an apron 20 times… It takes time that I can’t afford to spend…” (I14)*

In all three countries, another reason for low compliance with the PPE use was related to the limited cooperation from patients who failed to identify themselves as flu patients. PCPs described examples of consultations when patients started to talk about their symptoms during the consultation. At this stage, PCPs felt, it was late, and also inconvenient to put on PPE:

*“…I felt it would be ridiculous to put on a mask after he is already in my room and he was sitting for maybe half an hour in the waiting room…” (I6)*

In Israel, disinclination to use PPE was particularly common among PCPs, and they indicated that patients if they were provided with masks, usually did not use them too:

*“No, I didn’t put on the mask… Patients could take masks, but they did not put them on…” (I8)*

In Australia, the burden of personal safety arrangements was especially pronounced in the context of PPE supply. Australian health authorities expected PCPs to stockpile PPE in the event of a pandemic. PCPs, however, were not prepared to stockpile PPE more than *“a core stock for our staff”(M19)* as they were reluctant to part-fund the public health response:

*“… that becomes not an issue of primary healthcare, why we should be bearing the cost of it (PPE)? Cost for us is a major issue.” (M19)*

### Communication with the health authorities

Communication with the health authorities was frequently criticised by PCPs from the three countries. Two types of communication problems were raised: information flow from the health authorities to update PCPs about the latest guidelines, and communication from PCPs to report about the suspected cases or to provide feedback about the policies.

#### Communication of policies and guidelines to PCPs

PCPs from the three countries described the communication of policies to PCPs as “*not synchronous with the on-the-ground experience*”*(M2*), guidelines being lengthy, not oriented to primary care, too frequently updated. Many complained that critical updates were published in the media before they were sent to PCPs by the health authorities. This situation was described by one doctor as *“playing catch-up with the popular media all the time”(M16)* and some PCPs felt that it challenged the patients’ trust in their doctor as a source of reliable information.

In Australia and England, PCPs also reported communication being redundant and sometimes conflicting as the updates were available from “*a variety of sources, not one single source that was the authoritative voice.”(L11).*

#### Bottom-up communication from PCPs

In Australia and England, early involvement of PCPs in the pandemic management implied that they were the main source of surveillance for the health authorities, and they were required to report every suspected case. The reporting was mandatory for arranging the virological test and prescription of antivirals. This reporting was described by PCPs from these thwo countries as being time-consuming and hard to perform in primary care:

*“…you had to wait 45 min on the phone to get an approval number … and without that approval you couldn’t initiate the treatment.” (M2)*

In Israel, the opinion of PCPs was particularly negative regarding health authorities not being using their expertise to receive feedback about the situation on the coalface and that *“there was not enough dialogue with primary care physicians”(I19)*. They believed that they could contribute to improve the case definition sensitivity early in the pandemic, to provide feedback about the severity of the disease and the applicability of the guidelines to the reality of primary care. Many felt resentment for not being able to influence decision-making in a field that directly related to their professional activity:

*“…if primary care physicians are not convinced, the message is not passed to the public” (I14)*

## Discussion

The data concerning perceived difficulties of the pandemic response in primary care identified challenges specific to the pandemic situation in each of the three studies countries. These challenges were influenced by the timing and severity of the disease spread, level of PCP involvement in the response, support provided to PCPs by the health authorities, and organization of primary care services in a country.

### Challenges for the Australian approach to PCP involvement in pandemic management

Appearance of the pandemic flu in Australia in May 2009, against the background of autumn Influenza-like Illnesses (ILI) [17], put PCPs at a disadvantage compared to their counterparts in Israel and England where the disease occurred outside the regular flu season [18, 19]. ILI in Australia peaked in mid-July and early August, after which they gradually decreased, reaching normal spring seasonal rate by mid-October [17]. The low sensitivity of the case definition during the early stages of the pandemic paved the way for the transmission of the disease in the community [1] and resulted in a short and very intensive containment phase.

Primary care clinics were supposed to develop and implement pandemic management plans for their clinics according to the workplan kits provided by the authorities in each Australian State or Territory [6]. Primary care clinics were in charge of preparing a triage plan for the suspected cases, introducing infection control measures and reporting the suspected cases to the health authorities [20].

The first crucial challenge described by Australian PCPs was the workload associated with the large flow of patients. During the containment phase, the surge of the pandemic flu patients was concurrent with the surge of the worried-well, both appearing against the background of patients with other winter infections. This workload was aggravated by the patients’ demand for personalized advice, despite the fact that the authorities provided comprehensive general information to the public about the disease, infection control and treatment. This revealed the tension between the public health approach in provision of information to the public and guidelines to PCPs, and personalized care that the patients expected to receive from PCPs.

The next crucial issue was practice reorganization in order to introduce infection control measures. These measures necessitated introduction of additional procedures in primary care clinics, such as patient segregation. In Australia, PCPs were expected by the health authorities to reorganize their practices in such a way that they would become responsive to the pandemic.

Australian PCPs experienced difficulties reorganizing their clinics to introduce infection control measures as the main orientation of modern primary care is on chronic care delivery, given the increasing prevalence of patients with chronic diseases and multimorbidity in general practices [21, 22]. The usual barriers stated were the limited space in clinics and difficulty in room occupancy reorganization.

Reporting of the surveillance data for the public health authorities during the early stages of the pandemic presented an additional challenge. Australian PCPs were asked to report every suspected patient to local Public Health Units in order to arrange for the viral test and antiviral drugs. Our data confirmed the finding of other studies regarding the time consuming nature of the surveillance reporting during a pandemic response [1, 23]. This study adds further that PCPs felt this compulsory reporting (before taking the swab test and prescribing antiviral drugs) represented a lack of trust from the health authorities in their professional decision making and intrusion into their clinical autonomy. The unsettling feeling that they were not able to provide clinical treatment for patients without the permission of a public health officer was the dominant theme for Australian PCPs, and it represents the clash of responsibilities in the face of poor role delineation.

Personal safety was the next crucial issue for Australian PCPs. Barriers for stockpiling PPE and antivirals for prophylactic treatment of the staff in primary care were reported in previously conducted studies concerning pandemic preparedness in primary care [24, 25]. The challenge of the PPE access during a disease outbreak, in terms of its cost and shortage, was also reported previously in relation to the SARS [26–28]. At the beginning of the pandemic, the health authorities expected PCPs to purchase PPE “through normal suppliers”[29]. PPE was part of the National Medical Stockpile in Australia but it was only made available to PCPs from this source later on in the outbreak [30]. Our results confirmed the finding of previous reports that PCPs saw the supply of PPE and prophylactic treatment as the responsibility of the health authorities [24, 25], and suggested that the PCPs’ response demonstrated their reluctance to bear the costs of the public health response.

### Challenges for the English approach to PCP involvement in pandemic management

In England, the disease appeared outside the regular flu season and the pandemic flu spread was affected by school closure over the summer break [31]. The disease spread in two waves – mid-July and September-December [19].

In England, the approach was to avoid primary care clinic attendance by the suspected patients during the whole pandemic period. Throughout the containment phase, this was achieved by the strategy of assessing the suspected patients during home visits. While home visits were a routine practice and an important component of the workload in primary care in England in the past [32], today they are reported to represent about 4 % of all consultations [33]. Our data elicited the challenges of implementing this approach in primary care during pandemics. The burden of workload associated with home visits, phone consultations and surveillance reporting to the health authorities was evident from the interviews with English PCPs.

When it was perceived that the pressure of home visits became unbearable, the NPFS was organized by the Department of Health [34]. The approach of the supported self-care and wide distribution of the antivirals to symptomatic flu patients was part of the pre-pandemic planning for primary care in England [35]. This, indeed, resulted in a reduction in PCPs’ consultations [36, 37]. The data from the interviews showed that this approach was generally welcomed by PCPs, which is consistent with the findings of a cross-sectional survey conducted in the UK [38]. However, this in turn generated further concerns about the safety of phone consultations performed by non-clinical staff, that were also reported in other studies [38, 39].

PCPs from England indicated that they consulted many patients who needed personalized clinic advice. This is despite the extensive information provided by the NPFS. The possible explanation for this phenomenon is that the provision of information from the official sources addressed the cognitive risk judgment of the population but not the “emotional” concerns. These were found to be significant predictors of behavioural responses during the initial stages of the 2009/A/H1N1 outbreak [40]. Also, it was reported that while about 90 % of members of the public across the UK were satisfied with the amount of information about the 2009/A/H1N1, 37 % still had information they wanted to know [41]. Between the additional information that the public wanted to know, the most common types were details on symptoms, and advice on prevention and treatment [41]. Since these pieces of information represented concerns that are usually addressed during primary care consultations, this may provide an explanation for the high presentation rate of patients seeking personalized advice.

### Challenges for the Israeli approach to PCP involvement in pandemic management

As in England, in Israel, the influenza pandemic occurred outside the regular flu season, which facilitated the detection of the suspected cases. The disease spread in three escalating waves – at the beginning of August, mid-September and mid-November [18], and was much longer compared to regular flu season in previous years [42]. The efforts to contain the disease in Israel were facilitated by the school closure over the summer break and autumn festive season [31, 42].

The Israeli policy of treating all suspected cases in hospitals at the beginning of the outbreak decreased the risk to PCPs of exposure to the potentially virulent virus, and freed them from the workload associated with the reporting of the cases to the health authorities and patient segregation. PCPs started to assess flu patients when the disease became widespread in the community. By this time, the information about the overall mild nature of the virus became available from countries where the first wave of the disease has already passed, such as Australia.

The challenge of the increased workload when PCPs started to consult the flu patients was described. However, Israeli PCPs indicated that the surge of the anxiety in the community, characterised by increased consultations for people who were concerned they may get sick, finished before the main surge of pandemic flu patients.

Interestingly enough, Israeli PCPs expressed limited compliance, compared to their Australian counterparts, with the need to introduce infection control measures in the clinics, as they believed these measures would not be effective in a situation when the disease had spread in the community. Similarly, Israeli PCPs were not enthusiastic about the PPE use. In contrast to the situation in Australia, PPE was distributed to primary care clinics through HMOs free of charge, so the issues of cost and accessibility were not raised during the interviews. PCPs emphasised the inconvenience of PPE use in primary care in terms of rapport with patients and time needed to change protective gear between consultations.

Previously, the compliance with PPE use during SARS in Singapore was explained using the Becker Health Belief Model which acknowledges that changes in behaviour to reduce threat depend on the perceived vulnerability, severity, effectiveness and barriers [26]. In that study, PCPs from Singapore changed their behaviour to accommodate the supply problems and inconvenience of PPE use, as they believed that the effectiveness of PPE in decreasing the threat on their lives outweighed the barriers. It is likely that Israeli PCPs, who started to consult flu patients later in the pandemic, had a lower risk perception of the disease, compared to their Australian counterparts, which reduced the perceived usefulness of PPE and the infection control measures. Thus, Israeli PCPs were unwilling to overcome the barriers of making changes in their usual practice, as they did not see the need for these changes based on the prevailing epidemiology of the disease.

Compared to the data from the interviews in two other countries, it seems that Israeli PCPs were more critical of the health authorities for not involving them in the pandemic planning and decision making during the pandemic. They did not acknowledge the fact that they were protected during the containment phase of the disease because all the suspected cases were managed in the hospital, and many expressed the idea that the pandemic response of the health authorities was “over-kill”.

### Challenges shared by PCPs from all three countries

The data elicited generic challenges that were evidenced in each of the three countries. These generic challenges were related to perceived difficulties in following pandemic guidelines. The identified themes revealed that PCPs experienced difficulties in translating pandemic policies and guidelines into practice. These difficulties were consistent with the conceptual framework that explains barriers to clinical recommendations implementation [43]. This framework suggested that to conform with guidelines, physicians must be aware and be familiar with them and overcome barriers of negative attitude and external barriers.

#### Barriers affecting knowledge

In this study, all participants were aware of the pandemic guidelines early in the pandemic. Health authorities sent the alert documents to PCPs directly and through the mid-level organizations that duplicated or customized these alerts to local circumstances. No reports of limited guideline accessibility were found. However, some PCPs revealed that their familiarity with the guidelines was limited.

*High volume of information* and *lack of time* present barriers for clinical guideline adherence in general [43]. During the pandemic response, the lack of time presented an obstacle to keep with the pace of guideline updates. As the result, PCPs felt uncertain concerning whether they provided treatment according to the latest update.

*Multiple sources of information* challenged the effectiveness of the emergency communication with PCPs. PCPs received information from different organizations (national, state, mid-level authorities and professional organizations) and they reported that these communications were uncoordinated and confusing. This finding is consistent with the results of a recent study that redundant messages during emergency situations increase communication challenges [44].

*Pandemic guidelines not oriented to primary care* and lacking understanding of the primary clinics were reported by PCPs. A similar problem was found during the SARS outbreak in Hong Kong [45]. PCPs in this study especially highlighted the problematic applicability of the guidelines for infection control and personal safety measures. Our findings suggest that the guidelines to control infection transmission in primary care should take into consideration the infrastructure and resource constraints as well as the importance of personalized communication between PCPs and their patients.

#### PCP attitudes to pandemic guidelines

Another factor that complicated implementation of the pandemic guidelines was that PCPs harboured doubts about some of the pandemic policies. Specifically, limited agreement was reported concerning the policies for antivirals prescription. The low level of confidence in antiviral medication was not exclusive to PCPs and was also found among hospital health care workers [46]. Difficulties in treatment with an unfamiliar drug highlighted PCPs’ concerns about implementing public health policy in which they were not convinced, or which contradicted their clinical judgment.

Interviewed PCPs felt that they could improve the relevance of the guidelines to primary care by adjusting them to the severity of cases that they consult in primary care, as opposed to the cases that were admitted to the hospital. However, there was no established route to provide feedback about the pandemic policies.

#### Barriers affecting PCP behaviour

The ability of PCPs to implement pandemic policies was affected by the involvement of mass media in policy dissemination. As distinct from the regular clinical guideline communication, policy updates during the pandemic were happening in an atmosphere of constant media attention. This framed public response to the public health messages from the health authorities during the 2009/A/H1N1 pandemic.

Despite the extensive public health advice that was communicated by the health authorities through the mass media, the public’s concerns about the relevance of the communicated information to their own health was not satisfied. As the public see PCPs as a trusted source of information about their health [47], the patients went to the PCPs asking them to translate the public health information to the individual patient level. This quest of the public for personalized information increased primary care consultation rates which formed operational difficulties at the primary care level. Apart from the pressure of workload associated with increased consultation rates, PCPs in this study reported finding themselves in an awkward position when they were not provided with the timely information from the health authorities to respond to the concerns of their patients. In many instances, mass media was reporting about the policy changes before health authorities had sent the official updates to PCPs.

### Scope of the study

In analysing key aspects of the challenges for the pandemic response in primary care, this study is confined to the experience of PCPs who worked during the 2009/A/H1N1 pandemic in three countries: Australia, Israel and England. This study was designed to provide analytic generalizations and did not aim to draw quantitative inferences about the population of PCPs in large. The purpose of this study was to open up new ideas and to contribute new findings to the emerging field of the influenza pandemic preparedness.

## Conclusion

The experience of the 2009/A/H1N1 pandemic highlighted the centrality of primary care in the pandemic response. Despite intensive pre-pandemic planning, numerous barriers for implementation of the pandemic policies in primary care were identified. Investigation of three different approaches for involvement of PCPs in the pandemic management showed that none of these approaches worked smoothly. Each of the investigated approaches, as well as apprehension of the primary care response in general, presented a unique experience that is important to take on board in the evaluation of the pandemic response and planning for its improvement.
